# Amygdala substructure volumes in Major Depressive Disorder

**DOI:** 10.1016/j.nicl.2021.102781

**Published:** 2021-08-08

**Authors:** Darren Roddy, John R. Kelly, Chloë Farrell, Kelly Doolin, Elena Roman, Anurag Nasa, Thomas Frodl, Andrew Harkin, Shane O'Mara, Erik O'Hanlon, Veronica O'Keane

**Affiliations:** a Trinity College Institute of Neuroscience, Lloyd Building, Trinity College Dublin, Dublin 2, Ireland; b Department of Psychiatry and Psychotherapy, Otto von Guericke University Magdeburg, Magdeburg, Germany; c Department of Psychiatry, Royal College of Surgeons in Ireland, Dublin 2, Ireland

**Keywords:** Major Depressive Disorder, Amygdala, Centromedial nucleus, Cortisol awakening response, Hypothalamic-pituitary-adrenal axis, Freesurfer

## Abstract

The role of the amygdala in the experience of emotional states and stress is well established. Connections from the amygdala to the hypothalamus activate the hypothalamic–pituitaryadrenal (HPA) axis and the cortisol response. Previous studies have failed to find consistent whole amygdala volume changes in Major Depressive Disorder (MDD), but differences may exist at the smaller substructural level of the amygdala nuclei. High-resolution T1 and T2-weighted-fluid-attenuated inversion recovery MRIs were compared between 80 patients with MDD and 83 healthy controls (HC) using the automated amygdala substructure module in FreeSurfer 6.0. Volumetric assessments were performed for individual nuclei and three anatomico-functional composite groups of nuclei. Salivary cortisol awakening response (CAR), as a measure of HPA responsivity, was measured in a subset of patients. The right medial nucleus volume was larger in MDD compared to HC (p = 0.002). Increased right-left volume ratios were found in MDD for the whole amygdala (p = 0.004), the laterobasal composite (p = 0.009) and in the central (p = 0.003) and medial (p = 0.014) nuclei. The CAR was not significantly different between MDD and HC. Within the MDD group the left corticoamygdaloid transition area was inversely correlated with the CAR, as measured by area under the curve (AUCg) (p ≤ 0.0001). In conclusion, our study found larger right medial nuclei volumes in MDD compared to HC and relatively increased right compared to left whole and substructure volume ratios in MDD. The results suggest that amygdala substructure volumes may be involved in the pathophysiology of state depression.

## Highlights

• MDD is associated with larger right sided medial nuclei amygdala volumes.
• MDD is associated with increased right:left whole and substructural volume ratios.
• MDD cortisol inversely correlated with left corticoamygdaloid transition area.
• The study implies the potential importance of amygdala substructure volumes in MDD.

## 1 Introduction

The defining experience in Major Depressive Disorder (MDD) is of an emotional change where the individual becomes persistently sad and/or unable to experience pleasure. The experiential aspects of MDD theoretically map onto brain limbic circuitry, in particular the subcortical temporal lobe limbic structures of the hippocampus and the amygdala (Kaiser et al., 2015, Pruessner et al., 2010). Evidence indicates that state depression is associated with reduced hippocampal volumes (Brown et al., 2014, Nolan et al., 2020, Schmaal et al., 2016, Stephanie Campbell et al., 2004, Wise et al., 2017). Hippocampal pathology is consistent with the key cognitive difficulties experienced in MDD, such as impairments in short term and autobiographical memory formation (Kohler et al., 2015) and attentional problems (Disner et al., 2011).

In a previous study we replicated the common finding of smaller hippocampal volumes in MDD, and also that the core hippocampal neuronal subfields involved in coding biographical memories – in particular, the left cornu ammonis (CA1-CA4), and the dentate gyrus were relatively reduced in an MDD (Doolin et al., 2018, Roddy et al., 2019). This implies that hippocampal substructure volumes, rather than whole hippocampal volumes, may yield more precise information on the potential brain pathology in MDD. Core subfields volume reduction at the center of the hippocampal hub increased with chronicity of MDD, with CA1 volume being a predictor of depression (Roddy and O'Keane, 2019, Roddy et al., 2019). The latter finding suggests a disease process in the hippocampus of those suffering from more severe depressive illness.

The amygdala, tightly nestled on top of and densely interconnected with the hippocampus, plays an essential role in emotional processing, affective/mood state, fear conditioning and extinction and social behaviours (Fox and Shackman, 2019, Hur et al., 2019, Janak and Tye, 2015, Lindquist et al., 2012, Putnam and Chang, 2021, Shackman et al., 2016). In contrast to the more reliable decrease in hippocampal volumes in MDD, studies investigating amygdalae volumes are inconsistent (Schmaal et al., 2020). Studies show reduced (Ancelin et al., 2019, Bora et al., 2012, Kronenberg et al., 2009, Schuhmacher et al., 2012, Tang et al., 2007), increased (Frodl et al., 2002, Frodl et al., 2003, Saleh et al., 2012, van Eijndhoven et al., 2009, van Elst et al., 2000), and unchanged amygdala volumes in MDD (Arnone et al., 2012, Brown et al., 2019, Shen et al., 2017, Stephanie Campbell et al., 2004). Inconsistencies may reflect sample heterogeneity, confounding factors and methodological differences (Hamilton et al., 2008, Saygin et al., 2017).

**Fig. 1:**
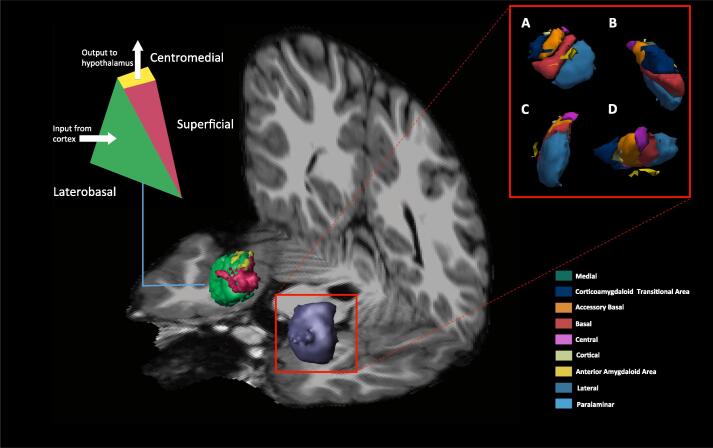
**Whole amygdala and amygdalar subfields.** Representative oblique T1 image showing the left whole amygdala (purple) and right amygdala divided into the laterobasal (green), centromedial (yellow) and superficial (red) clusters of nuclei. The right amygdala is expanded out to show all nine computed nuclei from four views; A, frontal; B, medial; C, lateral; D, superior. Different colours represent specific nuclei; green, medial; dark blue, corticoamygdaloid transitional area; orange, accessory basal; red, basal; purple, central; off-white, cortical; yellow, anterior amygdaloid area; light blue, lateral; turquoise, paralaminar. The left nuclear groups are colour coded and represented diagrammatically to show amygdalar input and output pathways. (For interpretation of the references to colour in this figure legend, the reader is referred to the web version of this article.)

Like the hippocampus, rather than being a unitary structure, the amygdala is formed from a collection of interconnected substructures (nuclei) that relay signals from multiple brain areas (Fig. 1, Table S1). Amygdala nuclei can be clustered into three anatomico-functional groups; laterobasal, centromedial and superficial (Table 2) (Heimer et al., 1999). Broadly speaking, the main cortical and sensory inputs enter the amygdala through the laterobasal nuclei, where they are returned to their cortical areas of origin (Freese and Amaral, 2005). The superficial nuclei are important in affective, memory and social processing and have widespread connections to other limbic structures (Goossens et al., 2009). Consolidated outputs from the amygdala emerge primarily from the medial structures, i.e. the medial and central nuclei (Table S1) (Heimer et al., 1999), and this area of the amygdala is found to be particularly activated following negative emotional stimuli (Hrybouski et al., 2016).

The ventral amygdalofugal pathway, anterior commissure and stria terminalis are the three main efferent pathways from the amygdala (Kamali et al., 2016). The stria terminalis and amygdalofugal pathways emerging from the centromedial nuclei travel to the emotion-making and homeostatic stress systems in the hypothalamus (Heimer et al., 1999). Projections from the bed nuclei of stria terminalis to the lateral hypothalamus and the ventral tegmental area have been shown to modulate divergent emotional/physiological states (Giardino et al., 2018, Jennings et al., 2013).

An anxiety-generating circuit from the central amygdala to CRH-secreting neurons in the dorsolateral stria terminalis has been identified in animal studies (Kalin et al., 2016, Kovner et al., 2020, Pomrenze et al., 2019) and the central amygdala is involved in social and non-social threat processing (Andraka et al., 2021). The medial amygdala is also implicated in complex social behaviors (Hu et al., 2021, Kwon et al., 2021, Shemesh et al., 2016). Similar to the hippocampus, glucocorticoid receptors are expressed in the amygdala (Wang et al., 2014) but, in contrast to the hippocampal feedback, amygdalar drive promotes hypothalamic CRH secretion (Herman et al., 2016, Myers et al., 2012), mainly mediated by centromedial nuclei (Herman et al., 2016). This is of relevance to MDD because overdrive in the hypothalamic–pituitary-adrenal (HPA) axis has been established in subgroups of MDD (Doolin et al., 2017, Stetler and Miller, 2011).

Several studies have investigated whole amygdala volumes and HPA axis reactivity in MDD (Kronenberg et al., 2009, Schuhmacher et al., 2012) and HCs (Barry et al., 2017) and the findings are mixed. Schuhmacher and colleagues (Schuhmacher et al., 2012) reported that larger left and right amygdala volumes at baseline positively correlated with normalisation of HPA response to the dexamethasone-corticotrophic releasing hormone challenge (Dex/CRH) following antidepressant treatment in a subgroup of inpatients with recurrent depressive disorder.

Elevated maternal cortisol levels during gestation were associated with larger right amygdala volumes (Buss et al., 2012) and increased amygdala functional connectivity in school-age girls, and may be related to a higher level of affective problems (Graham et al., 2019). Enlargement of the basolateral amygdala has been linked to childhood anxiety (Qin et al., 2014), whereas other studies in children have shown associations between greater cortisol stress responses and reduced amygdala volumes (Fowler et al., 2021, Pagliaccio et al., 2014). In a subgroup of young healthy adults exposed to maternal postnatal depression in early life, right hemisphere amygdala volume was reported to be negatively correlated with cortisol reactivity in response to the Trier Social Stress Test (Barry et al., 2017). In healthy adults, cortisol responses to the Dex/CRH test (Kiem et al., 2013) and acute social stress (Vaisvaser et al., 2013) were found to be inversely correlated with amygdala-hippocampal functional connectivity (FC). Greater cortisol levels have been reported to be correlated with increased amygdala-centred FC in responses to fearful faces (Hakamata et al., 2017), and in young adults with a history of depression, cortisol levels correlated with increased amygdala connectivity to cognitive control network regions (Peters et al., 2019).

Precise measurement of the amygdala and its composite nuclei is now possible using Freesurfer 6.0 software that provides objective detailed automated segmentation measures (Saygin et al., 2017). A 7 Tesla MRI pilot study using this approach did not show any differences in amygdala nuclei volumes in 24 antidepressant free MDD participants compared to 20 matched healthy controls, though MDD severity as measured by the Montgomery-Asberg Depression Rating Scale (MADRS) was negatively correlated with multiple amygdala nuclei (Brown et al., 2019). The same group reported that MDD was associated with structural hyperconnectivity between the right lateral, basal, and central nuclei, and the rest of the brain, whereas the left medial nucleus showed significantly lower connection density compared to HC (Brown et al., 2020).

To date, no study has explored the relationship between amygdala subnuclei volumes and HPA axis reactivity in MDD. The aim of this study was to assess whole and functionally grouped amygdala nuclei volumes in MDD using automated segmentation and to explore the relationship between amygdala volumetrics and HPA axis function.

## 2 Methods

### 2.1 Participants and clinical data

Eighty people with Major Depressive Disorder (MDD) were compared to eighty-three HC without MDD. All participants completed the Structured Clinical Interview for DSM IV (SCID) (American Psychiatric Association, 1994) and Hamilton Depression Rating Scale (HAM-D) (Hamilton, 1960). Inclusion in the MDD group required a current SCID diagnosis of MDD and a HAM-D score of ≥ 17. Controls were required to have no active or previous SCID diagnosis and a HAM-D less than 8. Ethical approval was obtained from the Tallaght Hospital/St James Hospital Joint Research Ethics Committee, Dublin and fully written informed consent was obtained prior to enrolment. Full inclusion and exclusion criteria are described in Supplementary Information (SI).

### 2.2 MRI acquisition

All data was acquired on a Philips (Best, Netherlands) Intera Achieva 3.0 Tesla MR system (32-channel head coil) at Trinity College Institute of Neuroscience, Dublin.

• **T1**. 180 axial high-resolution T1-weighted anatomical images (T1W-IR1150 sequence, TE = 3.8 ms, TR = 8.4 ms, FOV 230 mm, 0.898 × 0.898 mm^2^, in-plane resolution, slice thickness 0.9 mm, flip angle alpha = 8°).
• **T2-**FLAIR. 60 axial T2-FLAIR images (TE = 120 ms, TR = 2800 ms, 0.49 mm × 0.49 mm in-plane resolution, slice thickness 3 mm, flip angle alpha = 8°).

### 2.3 Image Analyses

Cortical reconstruction and segmentation were performed using the Freesurfer 6.0 (http://surfer.nmr.mgh.harvard.edu/) with the hippocampus/amygdala module to extract amygdala measures (Fischl, 2012, Fischl and Dale, 2000). The technical details of these procedures are described elsewhere (Desikan et al., 2006, Fischl et al., 2002, Reuter et al., 2012). This module interrogates contrast differences between amygdala substructures using previously defined in-vivo and ex-vivo amygdala atlases to determine substructure characteristics. The procedure was optimized by combining T1 and T2-Flair inputs and selecting the 3 T MRI flag and multispectral segmentation in Freesurfer. Nine substructures were computed (Table 2). Full details on amygdala segmentation are described in SI.

### 2.4 Composite measures

Computed nuclei volumes were summed together to create composite measures based on neuroanatomical definitions. Three amygdala composites were generated to correspond with the three anatomic-functional groups: superficial, laterobasal and centromedial (Table 2) (Heimer et al., 1999, Johnston, 1923). A further whole amygdala volume was generated by summing all computed outputs.

### 2.5 Cortisol measures

Salivary cortisol was measured in a subset of 30 MDD patients and 25 HC. Saliva samples were collected by the study participants at three time points after wakening (0, 30, and 60 min) on the day prior to the scan, and samples were analysed by Liquid Chromatography-Mass Spectrometry (LC-MS). Participants were excluded if they did not complete all three samples. Laboratory analysis methods and subsequent CAR parameter calculations (areas under the curve with respect to increase and ground) are described in the SI.

### 2.6 Statistical Analyses

All extracted subfield volumes were systematically inspected visually and measures exported to SPSS26 (https://www.ibm.com/analytics/us/en/technology/spss/). Mixed-method-Analyses of Variance (ANOVA) were used to investigate group-wise differences in substructure/composite volumes and across all substructures/composites and hemisphere (left/right) of the amygdalae. To clarify any driving effects identified, additional post-hoc Analyses of Covariance (ANCOVA) were used to compare between-group differences (Controls vs MDD) for each substructure/composite and hemisphere independently. Age, sex and estimated total intracranial volume (eTIV) were entered as covariates throughout. The Benjamini-Hochberg procedure was used to correct for multiple comparisons (Benjamini, 2010).

Partial correlations were performed to examine the relationship between nuclei and composite amygdalar measures, and HAM-D scores within the MDD. The right: left volume (R-L) ratios were non-normally distributed and were assessed using log transformed delta volume differences via a series of nuclei/composite independent ANCOVA’s. Partial correlations were used to explore the relationship between the CAR and amygdala nuclei and composites in a subset of participants.

## 3 Results

### 3.1 Demographics

There were no differences between controls and MDD patients for age, sex, or handedness (Table 1). 87% of MDD patients were taking antidepressants. There were no demographic differences between the CAR subsamples or between these subsets and the total group (Table S7).

**Table 1:** Demographic data for complete sample.

	Con N = 83	MDD N = 80	Con v MDD *p*-value
Age: Mean (SEM)	31.5 (1.4)	34.5 (1.4)	0.13
Range (years)	16–64	17–64	–
Male/Female (%M)	34/49, 41%	23/57, 29%	*X*^2^: 0.14
Handedness (R/L)	75/8	73/7	*X*^2:^ 1.0
HAMD (SEM)	1.3 (0.4)	22.2 (0.4)	1.10E-75
MDD duration months	–	29 (4)	–

Con, controls; Dep, depressed; HAM-D, Hamilton Depression scale; M, male; MDD, major depressive disorder; R/L, right or left handed; SEM, standard error of the mean. Age and HAMD analysed using t-tests, male/female and handedness analysed by chi square tests.

### 3.2 Amygdala volumetrics

The initial global ANOVA generated no significant main effect for group. There was no main effect for hemisphere, but a significant group × hemisphere interaction (Greenhouse-Geisser) [F(1dof, 7.11), p = 0.009, partial eta^2^ = 0.05, power = 0.75] was found. A main effect for substructure [F(8dof, 14.42), p = 0.00001, partial eta^2^ = 0.05, power = 0.75] and group × hemisphere × substructure interaction [F(2.318dof, 4.37), p = 0.01, partial eta^2^ = 0.03, power = 0.75] was identified (Table S2).

Post-hoc ANCOVAs following FDR correction for each independent nucleus revealed a larger right medial nucleus (p-uncorrected = 0.002) in MDD (Table 2). No correlations between HAM-D scores and amygdalar volumes survived FDR correction in the MDD group (Table S4a, b).

**Table 2:** Between group differences for individual amygdalar measures and R-L amygdalar ratios (log transformed).

Structure	MDD (mm^3^)	Controls (mm^3^)	Con v MDD	Con v MDD R-L Asymmetry
Nucleus/Composite measure	Mean (SEM, 95% CI)	Mean (SEM, 95% CI)	p-uncorrected	p-uncorrected
**LEFT**				
Lateral	643.53 (5.83, 632–655)	642.59 (5.79, 631–654)	0.91	0.25
Basal	417.06 (3.38, 410–424)	422.39 (3.40, 416–429)	0.27	*0.047*
Acc basal	252.31 (2.21, 248–257)	254.11 (2.12, 250–258)	0.57	0.076
AAA	52.70 (0.68, 51–54)	53.81 (0.67, 53–55)	0.25	0.815
Central	43.27 (0.79, 42–45)	45.40 (0.78, 44–47)	*0.059*	**0.003**
Medial	20.68 (0.64, 19–22)	19.70 (0.64, 18–21)	0.28	**0.014**
Cortical	24.97 (0.44, 24–26)	24.77 (0.44, 24–26)	0.75	0.239
CATA	192.07 (1.80, 189–196)	192 (1.8, 188–195)	0.82	0.855
Paralaminar	48.77 (0.52, 48–50)	50.52 (0.51, 50–52)	*0.018*	0.287
Whole amygdala	1692 (13, 1666–1718)	1699 (13, 1673–1725)	0.7	**0.004**
Laterobasal	1358 (11, 1337–1379)	1367 (11, 1346–1388)	0.57	**0.009**
Centromedial	64.25 (1.3, 62–67)	65.10 (1.29, 63–68)	0.65	0.107
Superficial	269.72 (2.4, 265–274)	269.86 (2.4, 265–275)	0.97	0.164

**RIGHT**				
Lateral	668.18 (5.05, 658–678)	652.81 (5.02, 643–663)	*0.033*	
Basal	434.95 (3.18, 429–441)	430.91 (3.20, 425–437)	0.37	
Acc basal	261.46 (2.20, 257–266)	259.69 (2.12, 255–264)	0.57	
AAA	56.73 (0.75, 55–58)	56.30 (0.74, 55–58)	0.69	
Central	48.86 (0.96, 47–51)	46.58 (0.94, 45–48)	0.095	
Medial	23.40 (0.61, 22–25)	20.64 (0.60, 20–22)	**0.002**	
Cortical	26.87 (0.41, 26–28)	26.96 (0.41, 26–28)	0.87	
CATA	193.30 (1.74, 190–197)	193.00 (1.72, 190–196)	0.87	
Paralaminar	50.84 (0.44, 50–52)	50.93 (0.43, 50–52)	0.88	
Whole amygdala	1760 (12, 1736–1785)	1734 (12, 1710–1758)	0.13	
Laterobasal	1412 (10, 1393–1431)	1392 (10, 1373–1412)	0.16	
Centromedial	72.33 (1.42, 70–75)	67.11 (1.40, 64–70)	*0.014*	
Superficial	277.20 (2.4, 273–282)	275.64 (2.4, 271–280)	0.65	

Between group differences for individual amygdalar measures and R-L amygdalar ratios following ANCOVA correcting for age, sex and eTIV. Correction for multiple comparisons using the Benjamini-Hochberg procedure determined a significance value of p ≤ 0.002 for 18 nuclei/composites (9 left and right) with ‘Con v MDD’ and p ≤ 0.014 for R-L asymmetry. Bold text survives FDR correction. Italic text denotes p ≤ 0.05 but not surviving the FDR threshold. For details of nuclei and composites see Table S1. AAA, anterior amygdalar area; ANCOVA, analysis of covariance; CATA, cortical amygdalar transition area; FDR, false discovery rate; MDD, major depressive disorder; R-L, right-left; SEM, standard error of the mean.

Hemisphere asymmetry ANCOVAs revealed significantly increased R:L ratios in the whole amygdala (p-uncorrected = 0.004), laterobasal composite (p-uncorrected = 0.009), central (p-uncorrected = 0.003) and medial nucleus (p-uncorrected = 0.014) volumes in MDD compared to controls (Table 2).

### 3.3 Cortisol awakening response

There were no significant differences in cortisol measures between the MDD and control groups (Table S8). Within the MDD group, the CAR, as measured by the AUCg was negatively correlated with the left cortical amygdalar transition area (Table 3, Fig. S1a). No correlations were found between any CAR and amygdala volumes in the HC group (Table S10).

**Table 3:** Cortisol partial correlations in the MDD group.

Structure	T0 min	T30 min	T60 min	AUCg
Nucleus/Composite measure	p-uncorrected, value (r-statistic)			
**LEFT**				
Lateral	0.507 (−0.136)	*0.024* (−0.441)	*0.025* (−0.437)	*0.015* (−0.474)
Basal	0.953 (−0.012)	0.198 (−0.261)	0.127 (−0.307)	0.104 (−0.326)
Acc basal	0.656 (−0.092)	0.936 (0.017)	0.459 (−0.152)	0.29 (−0.216)
AAA	0.407 (−0.173)	0.072 (−0.366)	0.076 (−0.362)	0.069 (−0.37)
Central	0.897 (−0.027)	0.899 (0.027)	0.611 (0.107)	0.536 (0.13)
Medial	0.767 (−0.064)	0.476 (0.153)	0.571 (0.122)	0.91 (−0.024)
Cortical	0.999 (0)	0.434 (0.164)	0.992 (0.002)	0.857 (−0.038)
CATA	*0.006* (−0.524)	*0.03* (−0.426)	**0.000130** (−0.681)	**0.00003** (−0.72)
Paralaminar	0.958 (0.011)	0.415 (−0.167)	0.346 (−0.193)	0.336 (−0.197)
Whole amygdala	0.469 (−0.148)	0.115 (−0.317)	*0.04* (−0.405)	*0.022* (−0.446)
Laterobasal	0.645 (−0.095)	0.096 (−0.333)	0.055 (−0.38)	0.033 (−0.419)
Centromedial	0.854 (−0.039)	0.456 (0.156)	0.515 (0.136)	0.675 (0.088)
Superficial	*0.045* (−0.396)	0.07 (−0.361)	*0.002* (−0.579)	*0.001* (−0.624)

**RIGHT**				
Lateral	0.388 (−0.18)	0.353 (−0.194)	0.321 (−0.207)	0.356 (−0.193)
Basal	0.259 (−0.23)	0.055 (−0.381)	0.093 (−0.336)	0.1 (−0.33)
Acc basal	0.141 (−0.297)	0.168 (−0.279)	0.328 (−0.2)	0.145 (−0.294)
AAA	0.828 (−0.045)	0.398 (−0.173)	0.984 (−0.004)	0.671 (0.088)
Central	0.848 (0.04)	0.755 (0.064)	0.601 (0.108)	0.484 (0.144)
Medial	0.21 (0.254)	0.132 (0.304)	*0.014* (0.477)	0.083 (0.347)
Cortical	0.994 (−0.001)	0.896 (−0.027)	0.874 (−0.033)	0.624 (−0.101)
CATA	*0.031* (−0.424)	*0.026* (−0.437)	*0.025* (−0.44)	*0.005* (−0.538)
Paralaminar	0.318 (−0.204)	0.098 (−0.332)	0.097 (−0.333)	0.169 (−0.278)
Whole amygdala	0.374 (−0.186)	0.336 (−0.201)	0.395 (−0.178)	0.357 (−0.192)
Laterobasal	0.336 (−0.201)	0.266 (−0.231)	0.281 (−0.224)	0.271 (−0.229)
Centromedial	0.49 (0.142)	0.36 (0.187)	0.14 (0.297)	0.196 (0.262)
Superficial	0.116 (−0.316)	*0.054* (−0.383)	0.116 (−0.316)	0.067 (−0.364)

Salivary cortisol was measured at 0, 30 and 60 min after waking. The cortisol awakening response was calculated using the area under the curve with respect to ground (AUCg). The FDR based threshold for significance was p ≤ 0.0003, marked in bold text. Italic text denotes p-values ≤ 0.05 but not surviving the FDR threshold. AAA, anterior amygdalar area; AUCg, area under the curve with respect to ground; CATA, cortical amygdalar transition area; FDR, false discovery rate; MDD, major depressive disorder.

## 4 Discussion

Using advanced automated segmentation, we compared the volumes of individual nuclei and composite groups of the amygdalae in patients with MDD and HC. The main finding was that the right medial nucleus was larger in the MDD group. Laterality differences were uncovered in whole amygdala volumes between the MDD and the HC groups, with the depressed group having an increased right-left ratio. This right amygdalar dominance in MDD was noticeably driven by increased volumes in the main amygdalar output structures on the right side, the central and medial nuclei, and the laterobasal group. Although cortisol stress responses were not statistically different between MDD and controls, there was an inverse relationship between the CAR as measured by the AUCg and the left corticoamygdaloid transition area (CATA) volume.

The absence of whole amygdala volume differences in MDD in this study is consistent with neuroimaging studies that have reported no overall changes in whole amygdala volumes (Arnone et al., 2012, Brown et al., 2019, Stephanie Campbell et al., 2004). Deeper substructural analysis investigating the constituent parts of the amygdala, i.e., the nine amygdalar nuclei and three composite clusters (laterobasal, centromedial and superficial groups), demonstrated a clear increase in the right medial nuclei volumes in MDD (Table 2). The centromedial group consists of the principal output nuclei to the hypothalamus (Barbier et al., 2018, Van de Kar and Blair, 1999) and have been shown to be particularly sensitive to negative emotional stimuli in HC (Hrybouski et al., 2016).

**Fig. 2:**
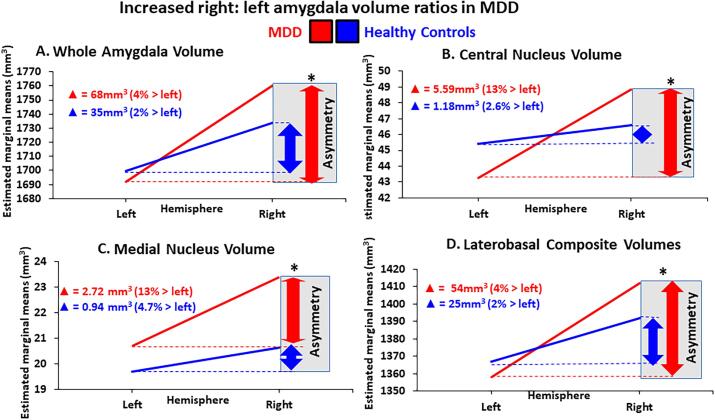
**Increased right: left amygdala ratios in MDD compared to controls.** The major depressive disorder (MDD) group (red) exhibited a greater right:left ratio compared to healthy controls (blue) in (**A**) whole amygdala volumes (p = 0.004); at the level of the (**B**) right central nucleus (p = 0.003), and the **(C)** right medial nucleus (p = 0.014); and at the composite level of the **(D)** laterobasal group (p = 0.009) (log transformed▲delta (%) = difference between right and left volume/left volume *100, *survives FDR significance threshold). (For interpretation of the references to colour in this figure legend, the reader is referred to the web version of this article.)

Although structural amygdala asymmetry is recognized in healthy humans (Pedraza et al., 2004), we found that there was a marked and significant increase in right compared to left volume measures in the MDD group that was not present in the HC. In MDD the right whole amygdala and right laterobasal composite were significantly increased relative to the left (Table 2). At the level of the nuclei, greater right-left asymmetry was found exclusively in the central and medial nuclei in MDD (Fig. 2).

In contrast to our finding of larger right medial nucleus volumes in MDD compared to HCs, a recent pilot study by Brown and colleagues did not show any differences in amygdala nuclei volumes in MDD (Brown et al., 2019). There are several differences between the Brown et al study and our study which could account for the discrepancy in findings. Importantly, our study included 80 patients with MDD and 83 HC in contrast to 24 MDD patients and 20 HC. It is therefore possible that differences between the groups were not detected in the Brown et al study due to the small sample size. Unlike the Brown et al study where patients were antidepressant free, most of our sample of MDD participants were prescribed antidepressants, though all fulfilled criteria for moderate or severe MDD at the time of scanning. We acknowledge the potential confounding influence of antidepressant medication, however, differences between amygdala subnuclei volumes according to antidepressant treatment status did not survive correction for multiple comparisons in our MDD group (Table S6). Another difference between the studies relates to field-strength. The advantages of increased spatial resolution of the 7 T field-strength in Brown et al’s study may have limited their sample size. Moreover, there is precedence in using 3 T and Freesurfer 6.0 to investigate amygdala substructural volumes in clinical cohorts diagnosed with dementia (Bocchetta et al., 2019) and PTSD (Morey et al., 2020).

It is interesting to note that Brown and colleagues, while finding no differences in nuclei volumes between MDD and HC groups, demonstrated lateralization of amygdala white matter tracts in MDD in the form of structural hyperconnectivity between the right lateral, basal, and central nuclei and the rest of the brain, whereas the left medial nucleus showed significantly lower connection density (Brown et al., 2020). The increased connection density in the right lateral and basal nuclei was driven by the stria terminalis, whereas the right central nucleus was driven by the uncinate fasciculus (Brown et al., 2020). These results when considered in the context of our primary finding of a larger right medial nucleus and of relative increases in right compared to left amygdalar volumes in MDD, indicate the potential importance of right amygdala substructure volumes in the pathophysiology of MDD.

Both preclinical (Baker and Kim, 2004, Guadagno et al., 2020) and clinical data (Baas et al., 2004, Bruder et al., 2017) suggests lateralization of amygdala function. Electrical stimulation of the right hemisphere may produce more dysphoric/negative responses compared to the left (Lanteaume et al., 2006, Smith et al., 2006) and is involved in the rapid appraisal of aversive stimuli (Gläscher and Adolphs, 2003, Sergerie et al., 2008). The left amygdala, conversely, has been shown to be involved in generally slower emotional appraisals and with more positive emotion generation (Gläscher and Adolphs, 2003, Sergerie et al., 2008). In addition to modulating neuroendocrine responses, the amygdala modulates autonomic nervous system activity in the context of emotionally salient stimuli processing (Beissner et al., 2013, Gläscher and Adolphs, 2003). Interestingly, an electrical stimulation study of nine people with epilepsy showed that amygdala stimulation can modulate autonomic activity without eliciting concurrent subjective emotional responses, with the exception of one patient who reported marked fear and anxiety when the right amygdala was stimulated, in an area speculated to be close to the right central nucleus (Inman et al., 2018). Unilateral right sided Electroconvulsive therapy in a group of 14 antidepressant treated patients diagnosed with treatment resistant depression increased right corticoamygdaloid transition area (CATA), basal, and lateral amygdala nuclei volumes (Gryglewski et al., 2019).

It is well established that MDD is strongly associated with stressful life events. The mechanistic mediators underlying the interaction between stress and the structural development of the amygdala, include but are not exclusive to cortisol, Brain-derived neurotrophic factor (BDNF), glutamate and epigenetic mechanisms (McEwen et al., 2016). Preclinical data suggests that chronic stress may induce divergent changes in amygdala and hippocampal volumes, with enlargement of amygdala regions and reduction of hippocampal regions, largely through alterations in dendritic remodelling (Blugeot et al., 2011, Lakshminarasimhan and Chattarji, 2012, Mitra et al., 2005, Roozendaal et al., 2009, Vyas et al., 2002, Vyas et al., 2004). In our cohort of MDD participants, we also found divergent changes in amygdala and hippocampal volumes, as evidenced by smaller hippocampal substructures bilaterally, but more pronounced on the left (Roddy et al., 2019) and the present finding of larger right medial amygdala nuclei, known to modulate complex social behaviours in rodents (Hu et al., 2021, Kwon et al., 2021, Shemesh et al., 2016). We acknowledge the association between amygdala substructural volumes and phase and duration of illness, however in our MDD group these associations did not survive correction for multiple comparisons (Table S5a, b).

The reciprocal shaping of the HPA axis and limbic brain regions, under the influence of environmental stressors, especially during neurodevelopmental windows, is of particular importance for the individualized sensitivity to the development and trajectory of stress related disorders such as depression. Exposure to early stressful events may lead to increases in amygdala volumes (Tottenham et al., 2010). However, the process is dynamic and dependent on developmental stage, as highlighted by a recent longitudinal study showing a transition from blunted morning cortisol in childhood to heightened levels in late adolescence, associated with smaller amygdala volumes by adolescence in those that experienced early stressful events (VanTieghem et al., 2021). This may in part explain some of the discrepancies in other studies in younger cohorts that show that elevations in cortisol are associated with larger amygdala volumes (Buss et al., 2012), while others show smaller amygdala volumes are associated with greater cortisol stress responses (Fowler et al., 2021, Pagliaccio et al., 2014).

Our study extends the existing findings by exploring, for the first time, the relationship between HPA axis activity and amygdala subnuclei volumes in MDD. The CAR measures the reactivity of the HPA axis in response to the natural stress of waking (Farrell et al., 2018). Chronic HPA overactivity as demonstrated by a raised waking (baseline) cortisol and a reduced CAR is commonly found in depression (Doolin et al., 2017, Frodl and O'Keane, 2013, Huber et al., 2006, Juruena et al., 2018, O'Keane et al., 2012). In our sample, there were no statistically significant differences in the CAR between the MDD and HC groups (Table S8) which may be related to the limited sample size of the cortisol subgroup analysis. However, within the MDD group, while there were mostly negative CAR associations with multiple amygdala nuclei bilaterally (Table 3), only the inverse relationship between the AUCg and the left CATA survived FDR correction (Fig. S1a), which was not found in the HC group. The lack of CAR differences between MDD and HC, together with the inverse relationship between the CAR and the left CATA in the MDD group suggests that the disruption in the reciprocal relationship between the HPA axis and the amygdala in MDD is non-uniform across the amygdala nuclei.

The CATA is a zone of confluence of the medial basal, paralaminar and periamygdaloid areas. In primates, the CATA provides the main modulatory input to the lateral subdivision of the central nucleus and is a specific target for hippocampal inputs (Fudge et al., 2012, Fudge and Tucker, 2009), and together with hippocampal inputs to the adjacent paralaminar nucleus are implicated in contextual fear learning (deCampo and Fudge, 2012). Consistent with this, in rodents the CATA is indicated in the processing of olfactory information related to reproductive and defensive behaviors (Cádiz-Moretti et al., 2016, Kondoh et al., 2016). While our finding of an inverse relationship between the CAR and the left CATA in MDD is an important exploratory step to further elucidate the intricate bidirectional relationship between the HPA axis and amygdala subnuclei volumes, it should be interpreted with caution due to the limited sample size of the CAR subgroups. Nonetheless, it suggests that further exploration of this relationship in larger longitudinal samples could be helpful in understanding the neurophysiology of pathological emotional states.

There are preliminary indicators that a deeper understanding of the pathophysiology of MDD can lead to translational benefits. Decreased right basolateral amygdala gray matter density was positively correlated with reductions in perceived stress after an 8-week mindfulness-based stress reduction intervention in stressed healthy individuals (Hölzel et al., 2010). More recently, there is growing interest in real-time fMRI amygdala neurofeedback as a clinical tool (Tsuchiyagaito et al., 2021, Young et al., 2017, Zotev and Bodurka, 2020), which has been combined with substructural volume analyses. For example, left amygdala real-time fMRI neurofeedback training in Post-Traumatic Stress disorder (PTSD) resulted in volume increases in the left hippocampal CA1 head region (Misaki et al., 2021). Our study showing that MDD is associated with specific alterations in amygdala nuclei, together with exploratory CAR associations, may open up avenues for the further advance of precise-personalized treatment paradigms.

## 5 Limitations

Automated segmentation in neuroimaging analysis has been argued to have interpretation and validity issues compared to expert manual segmentation (Dill et al., 2015). On the other hand, manual measurements are costly and time consuming whereas automated segmentation allows large volumes of data, in this case 163 subjects yielded almost 3,000 individual nuclei, to be analyzed accurately. One could argue that an automated proforma is necessary to translate research findings into routine clinical tests. While 3 T MRI is effective, we acknowledge that access to 7 T would have delivered enhanced spatial resolution.

Only a subset of participants had cortisol samples that were suitable for inclusion in the analysis, due to non– or partial compliance with the salivary collection schedule. The study would have been strengthened by having more subjects in the HPA axis arm. We acknowledge the potential confounding influence of antidepressant medication (Hamilton et al., 2008), however, differences between amygdala subnuclei volumes according to antidepressant treatment status did not survive correction for multiple comparisons in our MDD group. We did not record antidepressant type or dose. We acknowledge the mediating influence of traumatic early life events (Gerritsen et al., 2017, VanTieghem et al., 2021) but we didn’t capture this data. Nor did we precisely capture the age of onset for the entire MDD group. We did not complete the SCID for axis 2 personality assessments.

Mood and anxiety are intrinsically linked. As discussed above, enlargement of the basolateral amygdala has been linked to childhood anxiety (Qin et al., 2014). Our study focussed on MDD and while participants may have exhibited anxiety symptoms, they did not meet the threshold for DSM IV diagnosis. In keeping with the overlap of mood, anxiety, and stress, it is interesting to note that a recent well-powered 3 T MRI study of another stress related disorder, PTSD in military veterans, showed larger left and right central, medial, and cortical amygdala nuclei compared to HC (Morey et al., 2020). This study also demonstrated smaller bilateral paralaminar and lateral nuclei in PTSD compared to HC (Morey et al., 2020). Indeed, in our study, the left paralaminar nuclei were smaller in the MDD group in the initial analysis, but this significance did not survive FDR correction. Limited longitudinal inferences can be drawn from our cross-sectional data. Ultimately, large-scale longitudinal studies that incorporate a dimensional approach will be required to definitively resolve these issues (McLean et al., 2020).

## 6 Conclusion

This study using automated segmentation to perform amygdalar substructural volumetric analysis, found larger medial subnuclei volumes on the right in MDD compared to HC, as well as relatively increased right compared to left whole and substructural volume ratios in MDD. This implies the potential importance of right amygdala substructure volumes in the pathophysiology of state depression. CAR responses were inversely related to the left corticoamygdaloid transition area volume in MDD.

## Author contributions

V.O’K, T.F, A.F, S.O’M conceived the study and edited the manuscript. D.R, E.K, A.N, C.F, K.D, and E.R collected and analysed the data and edited the manuscript. J.K analysed the data and edited the manuscript.

## Funding

This project was funded under by the Irish Health Research Board as part of the REDEEM (Research in Depression, Endocrinology, Epigenetics and neuroiMaging) study at Trinity College Institute of Neuroscience and the Department of Psychiatry, Trinity College Dublin. Grant code: 201651.12553. We also thank the Meath Foundation, Tallaght University Hospital, for the initial project funding. We acknowledge use of the facilities of the Clinical Research Centre in the RCSI Education and Research Centre and Trinity College High Performance Computing resources and infrastructure funded by Science Foundation Ireland.

## Declaration of Competing Interest

The authors declare that they have no known competing financial interests or personal relationships that could have appeared to influence the work reported in this paper.
