# Supplementary material for: Amygdala substructure volumes in Major Depressive Disorder
Source: Neuroimage Clin. 2021 Aug 8;31:102781. doi: 10.1016/j.nicl.2021.102781 (PMC8361319; doi:10.1016/j.nicl.2021.102781)
Supplement: Supplementary data 1 [file mmc1.docx]

**Amygdala substructure volumes in Major Depressive Disorder**

***Supplemental Information***

**Supplementary methods**

**Participants and Clinical Data**

Depressed patients were recruited from a psychiatric outpatient clinic at Tallaght Hospital, Tallaght, Dublin 24, Ireland. Patients were initially referred by either their GP or through the Emergency Department. All patients who presented to these clinics with depression were considered for the study. In total eighty-three patients were recruited. Patients were initially screened by a consultant psychiatrist (VO’K). Exclusion criteria for patient entry into the study included contraindications to MRI, active alcohol or substance abuse disorder (within last year), head trauma, and any significant acute or chronic illness including; cardiovascular, pulmonary, gastrointestinal, hepatic, renal, immunological and any other major concurrent illness.

Patients with a diagnosis of schizophrenia, schizoaffective or any other psychotic disorder were excluded. Healthy controls (HC) were recruited through an active database of willing participants and the same exclusion criteria applied. In total, eighty controls were recruited.

All participants underwent a structured clinical interview for DSM-IV (SCID) (American Psychiatric Association, 1994) and the Hamilton Depression Rating Scale (Hamilton, 1960) (HAM-D) at TCIN (Trinity College Institute of Neuroscience, Trinity College, Dublin, Ireland) prior to scanning. Entry criteria for patients were that they must have a current DSM-IV diagnosis of Major Depressive Disorder (MDD) with a HAM-D of 17 and above. Any patient that reached the threshold for any psychiatric disorder as per the SCID, other than MDD was excluded. A HAM-D score greater than 17 is considered moderately depressed, and a score greater than 22 is considered severely depressed (Williams, 2001). Entry criteria for HC consisted of an absence of both active or previous SCID diagnosis and a HAM-D less than 8 (considered not depressed). A consensus meeting between the consultant psychiatrist (VO’K) and the research team at Trinity College Institute of Neuroscience (TCIN) was held prior to every subject entry into the study.

Fully written informed consent using a detailed information sheet was obtained at the clinic during recruitment and also prior to scanning at TCIN. Patients were given a pack containing cortisol Salivette® tubes and a set of detailed instructions in clinic. Participants were shown how to collect saliva in the tubes and they were asked to complete the samples at home before the scan.

Ethical approval was obtained from the Tallaght Hospital/St James Hospital Joint Research Ethics Committee (REC). This REC operates on the principles of the Declaration of Helsinki. Fully written informed consent was obtained with a clearly laid out Patient Information Sheet.

**MRI acquisition and analysis**

All data was acquired on a Philips (Best, Netherlands) Intera Achieva 3.0 Tesla MR system (32-channel head coil) at Trinity College Institute of Neuroscience, Dublin. 180 axial high-resolution T1-weighted anatomical images were acquired (T1W-IR1150 sequence, TE = 3.8 ms, TR = 8.4 ms, FOV 230 mm, 0.898 x 0.898 mm^2^, in-plane resolution, slice thickness 0.9 mm, flip angle alpha = 8^o^).

T1 images were inspected manually to ensure there were no issues with corrupted data, subject movement artifacts or poor/failed acquisition. Cortical reconstruction and volumetric segmentation was performed with FreeSurfer 6.0 (<http://surfer.nmr.mgh.harvard.edu/>) (Fischl, 2012a; Fischl and Dale, 2000). FreeSurfer is a brain imaging software package designed specifically for analysing research MRI scan data (Fischl, 2012b). FreeSurfer 6.0 contains a newly updated hippocampal and amygdala analysis module (<http://freesurfer.net/fswiki/HippocampalSubfieldsAndNucleiOfAmygdala>) that was used for this analysis. This module was developed primarily by Juan Eugenio Iglesias of University College London in association with Massachusetts General Hospital (Iglesias et al., 2015; Saygin et al., 2017). The hippocampal-amygdala tool uses a probabilistic atlas build upon very high-resolution ex-vivo T1 MRI data (0.1-0.15 mm resolution; 0.13 mm average isotropic resolution) as well as more conventional (1 mm isotropic) in-vivo MRI datasets (Iglesias et al., 2015). The model uses a Bayesian inference algorithm (Van Leemput, 2009) to calculate the topology and reveal substructures depending on the distinct contrast differences of adjacent substructures.

The hippocampal-amygdala module is an evolution of the previous hippocampal-only module released initially with FreeSurfer 6.0. This in turn, is an improvement of the more rudimentary hippocampal solo module found in FreeSurfer 5.3, which as well as only calculating seven substructures, demonstrated well documented problems with specific subfield volume errors (Wisse et al., 2014). The most up to date version can discriminate up to thirteen hippocampal subfields and nine amygdalar nuclei (Table S1). Joint segmentation of the amygdala and hippocampus simultaneously guarantees that these structures do not overlap or leave gaps, ensuring a more consistent segmentation.

FreeSurfer requires MATLAB for operating the hippocampal-amygdala module. MATLAB (Matrix Laboratory) ([www.mathworks.com](http://www.mathworks.com)) is a proprietary programming language and multi-paradigm numerical computing environment developed by Mathworks Inc. and allows matrix manipulations, plotting of functions and data and implementation of algorithms (Sobie, 2011). Due to the large number of substructures (18 amygdalar nuclei) across 163 subjects = 2934 individual substructures, the data was processed through the high-performance computers at Trinity College Dublin.

FreeSurfer 6.0 can also generate an estimate of total intracranial volume (eTIV) using the T1 images. The technical details of the procedures involved in running FreeSurfer, generating eTIV and implementing the hippocampal-amygdala toolbox are described elsewhere (Dale et al., 1999; Desikan et al., 2006; Fischl et al., 2002; Fischl et al., 1999; Fischl et al., 2004; Reuter et al., 2012). FreeSurfer optimization parameters were employed to generate more accurate outputs. FreeSurfer outputs included both visual representations of the individual substructures as well as volumes in mm^3^ of each region.

Computed nuclei volumes were summed together to create composite measures. Three amygdala composites were generated to correspond with the three anatomic-functional groups; superficial, laterobasal and centromedial (Table S1) (Heimer et al., 1999; Johnston, 1923). A further whole amygdala volume was generated by summing all computed outputs.

**Table S1.** Computed Freesurfer amygdalar nuclei and composites.

| **Structure** | **Definition** | **Comments** |
| --- | --- | --- |
| Lateral | The largest single nucleus of the amygdala, and probably the easiest to discrimate. Classic wedge/triangular shape. Dorsolateral to the Basal nucleus along the AP extent of the amygdala. | Has 5 subnuclei; dorsal anterior, intermediate, ventral, dorsomedial and dorsolateral. Has the highest number of neurons in the human amygdala. Considered main input region. |
| Basal | Borders the Lateral nucleus ventromedially. | Sensory information conveyed here from the Lateral nuceus. The main target of afferent from the prefrontal cortex. |
| Accessory Basal | This lies ventral to the basal nucleus and adjacent to the hippocampus caudally. | Has 3 subdivisions; magnocellular, parvicellular and intermediate. |
| Paralaminar nuclei | Wedged inferior the Basal, Lateral and Cortico-amygdaloid transition areas. | Relatively expanded in humans and non-human primates. Site of plasticity of the amygdala. Dense serotonergic connections. |
| Central | Dorsal to the Accessory Basal nucleus. Often circular or oval in shape. | Key output substructure of amygdala circuitry, primarily to the hypothalamus. Four subdivisions. Considered part of the extended amygdala. |
| Medial | Visible along anteroposterior extend of amygdala, medially. Slim and elongated. | Has the highest number of neurons in the human amygdala. Four subdivisions. Considered part of the extended amygdala. Important output to hypothalamus. |
| Anterior Amygdala Area | The most anterior end of the amygdala. | Contains an abundance of grid, border and head direction cells. Important for scene recognition and visospatial processing. |
| Cortico-Amygdaloid Transition Area | The most medial border of the amygdala. | A confluence of the medial basal, paralaminar and periamygdaloid areas. |
| Cortical | Smallest nucleus, bordering the Accessory Basal medially. | The cortical surface (facing the medial surface of the temporal lobe) is often referred to the periamygdaloid area. Can be divided into anterior and poster cortical nuclei. |
|  |  |  |
| Whole amygdala | Lateral, Basal, Accessory Basal, Paralaminar, Central, Medial, Cortical, Anterior Amygdala Area, Cortico-Amygdaloid Transition Area. | Total combined nuclei of amygdala. |
|  |  |  |
| Laterobasal | Lateral, Basal, Accessory Basal, Paralaminar. | Largest nucear group. Cortical-like with reciprocal connections with the cortex. Input area. |
|  |  |  |
| Centromedial | Central, Medial. | Smallest nuclear group. Striatal-like with connections to hypothalamus. Output area. |
|  |  |  |
| Superficial | Cortical, Anterior Amygdala Area, Cortico-Amygdaloid Transition Area. | Olfactory-like region. Possibly concerned with social processing. |

AAA; anterior amygdaloid area; Acc Basal, accessory basal; CATA, cortical amygdalar transition area.

**Cortisol measures**

Salivary cortisol was measured in a subsample of participants. All subjects were invited to participate in salivary cortisol collection. Some declined, and some failed to complete the sample collection to satisfaction: e.g., returning two samples, or taking all the samples after getting up.

*Saliva Sample Collection*

Participants were given a pack containing cortisol Salivette® tubes and a set of detailed instructions in clinic. Participants were shown how to collect saliva in the tubes and they were asked to complete the samples at home on the day prior to the scan. Saliva samples were collected into Salivette® tubes (Sarstedt, Nümbrecht, Germany) by the study participant at 0, 30 and 60 min after waking. Salivette® tubes were centrifuged for 10min at 3000 rpm and room temperature to extract the saliva from the insert. The saliva was aliquoted and stored in microtubes at 80C until analysis.

*Saliva Analysis*

Saliva samples were assessed for cortisol and cortisone content by liquid chromatography-mass spectrometry (LCMS). Standard ELISA assays give a combined measure of both cortisol and cortisone. The LCMS method separates out cortisone from cortisol allowing the active cortisol, rather than both, to be measured. Samples were analysed at the University of Manchester in the Department of Clinical Biochemistry. A Shimadzu Prominence LC system (Shimadzu, Milton Keynes, UK) was used for chromatography. The eluate was injected directly into a Quattro MicroTM tandem mass spectrometer (Waters, Manchester, UK). MassLynx NT 3.5 software was used for system control and data processing. This software used the height of the detected peaks, 1/x weighting and linear least-squares regression to produce a standard curve to derive concentrations. The lower limits of detection and quantitation are 1 and 2 nmol/L, respectively. The coefficient of variation (CV) at 2 nmol/L concentration = 7.5% and the bias 26%. For further details see (Owen et al., 2010).

Data cleaning was used to eliminate values that were extreme outliers (3 x interquartile range). Cortisol awakening response (CAR) was calculated using two formulae; the area under the curve with respect to increase from baseline (AUCi) and the area under the curve with respect to ground (Fekedulegn et al., 2007).

**Statistics**

*Outliers*

In brief, all extracted volume measures were systematically inspected using the standard "Explore" tool within SPSS which plotted and subsequently examined for normality and outlier identification based on the SPSS outlier criteria (defined as data points falling more that 1.5 x interquartile range and extremes as 3 x interquartile range). The extreme and outlier data points were removed prior to statistical analysis.

*Primary ANOVA*

A primary mixed-model analysis of variance (ANOVA) was used to investigate group-wise differences in nuclei volumes across all substructures and hemisphere (left and right) of the amygdalae. This analysis was performed with the 9 substructure volumes and hemisphere (left and right) entered as within-subject factors and group (MDD versus HC) entered as the between-group factor. Age, sex and eTIV were entered as covariates. Greenhouse-Geisser correction (Greenhouse and Geisser, 1959) was applied for all tests and any significant main effects or significant interactions were further examined using independent post-hoc univariate analysis of covariance (ANCOVA) with age, sex and eTIV as nuisance factors.

*Secondary ANOVA*

A second mixed-model ANOVA was performed to investigate group-wise differences in volume of our defined amygdalar composite measures, the laterobasal, centromedial and superficial groups of nuclei. In this analyses, amygdala composite measures and hemisphere were entered as within-subject factors and group (total depressed versus controls) as the between-group factor. Age, gender and eTIV were entered as covariates in the model. Again, any significant main effect and interactions were further investigated using post-hoc ANCOVA with age, sex and eTIV as nuisance factors.

*ANCOVA*

In order to clarify the driving effects identified by any main effect or significant interaction from the ANOVAs, additional post-hoc ANCOVA were used to compare between-group differences for each amygdala substructure and hemisphere independently. Age, sex, and eTIV were entered as covariates. The same methodology was employed for the composite measures using a similar approach. Multiple Comparison Correction (MCC) was performed using False Discovery Rate (FDR) correction (Benjamini, 2010). FDR can be considered a more targeted form of MCC correction. Traditional Bonferroni correction "punishes" all input p-values equally, by dividing the p-value by the number of tests (Sedgwick, 2014), whereas Benjamini-Hochberg (as a way to control the FDR) "punishes" p-values accordingly to their ranking (Benjamini, 2010). In studies where there are only a few tests, either method can be used. However, in conditions where there are a large number of individual tests (e.g. in this study with 18 amygdalar substructures, 9 left and 9 right), a Bonferroni correction will produce false negatives i.e. it will discard true observations. As such FDR can be particularly good for exploratory studies like this.

*HAM-D correlations*

Partial correlations were performed to investigate associations between HAM-D score and amygdalar measures for each hemisphere in the MDD group.

*Amygdala R-L symmetry*

The R-L symmetry of all nuclei volumes were examined to assess potential group differences in each nucleus. Left/Right nuclei symmetry was looked at using log transformed delta volume differences via a series of nucleus independent ANCOVA’s correcting for age, sex and eTIV.

*Cortisol*

The cortisol awakening response (CAR) was measured by calculating the area under the curve with respect to increase from baseline and ground; AUCi and AUCg respectively (Doolin et al., 2017; Fekedulegn et al., 2007). AUCg is a measure of the cortisol increase from baseline cortisol values; and AUCi is a measure of cortisol increases from a theoretical point of cortisol=0 at baseline. AUCb refers to the area under the curve with respect to baseline (such that AUCg – AUCi = AUCb). Split-group partial correlations were performed to examine the relationship between nuclei volume measures and CAR measures for each hemisphere.

**SUPPLEMENTARY Results**

**Amygdala Volumetrics**

The primary global mixed-model repeated measures ANOVA generated no significant main effect for group. There was no main effect for hemisphere, but a significant group x hemisphere interaction (Greenhouse-Geisser, F (7.11,1dof), p=0.009, partial eta2=0.05, power=0.75) was found. A main effect for sub-nucleus (F 14.42 (8dof), p=0.00001, partial eta2=0.05, power=0.75) and group x hemisphere x substructure interaction (F 4.37 (2.318dof), p=0.01, partial eta2=0.03, power=0.75) was identified.

The secondary nuclei specific repeated-measures ANOVAs found no significance within subject effects. A significant between-subject effect was found for the medial nucleus F 5.7 (1dof), p=0.02. Significant interaction effects (group x nucleus x hemisphere) were found in lateral, basal, central and paralaminar nuclei. In addition, significant interactions were identified in the composite measures for the whole amygdala, LB and CM (see Table S2).

**Table S2.** Repeated measures ANOVA correcting for age, sex and eTIV.

| Amygdala Structure/ Composite | Within Subject Effect | Between Group Effect | Interaction (Group x Nucleus x Hemisphere) |
| --- | --- | --- | --- |
|  |  |  |  |
|  | F, p-value | | |
| Lateral | 0.01, 0.91 | 1.26, 0.26 | 7.4, 0.007 |
| Basal | 0.97, 0.33 | 0.02, 0.88 | 6.1, 0.015 |
| Accessory Basal | 0.20, 0.66 | 0.001, 0.99 | 1.88, 0.17 |
| AAA | 0.37, 0.55 | 0.14, 0.71 | 3.02, 0.08 |
| Central | 0.01, 0.91 | 0.01, 0.94 | 9.93, 0.002 |
| Medial | 2.12, 0.15 | 5.70, 0.02 | 4.58, 0.034 |
| Cortical | 0.27, 0.60 | 0.01, 0.92 | 0.24, 0.62 |
| CATA | 0.12, 0.73 | 0.05, 0.83 | 0.01, 0.95 |
| Paralaminar | 5.26, 0.02 | 2.30, 0.13 | 7.60, 0.007 |
| Whole amygdala | 0.13, 0.72 | 0.33, 0.60 | 6.62, 0.011 |
| Laterobasal | 0.18, 0.67 | 0.16, 0.69 | 7.58, 0.007 |
| Centromedial | 0.18, 0.68 | 1.72, 0.19 | 9.88, 0.002 |
| Superficial | 0.05, 0.83 | 0.05, 0.82 | 0.36, 0.55 |

AAA, anterior amygdaloid area; Acc Basal, accessory basal; CATA, cortico amygdaloid transition area; eTIV, estimated total intracranial volume

**Table S3.** Effects of sex on volumes.

Differences in volumes between male and female in each group. FDR threshold of p<0.0055. Bold text survives FDR correction. Italic text denotes p-values <0.05 but not surviving the FDR threshold.

|  | CONTROL | | DEPRESSED | |
| --- | --- | --- | --- | --- |
|  | LEFT | RIGHT | LEFT | RIGHT |
| Lateral nucleus | **<0.0001** | **0.0003** | *0.0274* | **0.0022** |
| Basal nucleus | **0.0016** | **0.0025** | 0.0333 | **0.0027** |
| Accessory basal nucleus | **0.0002** | **0.0003** | 0.1257 | *0.0213* |
| Anterior amygdaloid area | 0.1148 | 0.2566 | 0.0443 | 0.5151 |
| Central nucleus | **0.0005** | 0.0102 | 0.3321 | 0.5356 |
| Medial nucleus | 0.0817 | *0.019* | 0.6003 | 0.5495 |
| Cortical nucleus | **0.0048** | **0.0084** | 0.1688 | 0.9977 |
| Corticoamygdaloid transition area | *0.0148* | **0.0333** | 0.0113 | *0.0343* |
| Paralaminar nucleus | **0.0032** | 0.0128 | 0.1846 | **0.0032** |
| Whole amygdala | **0.0002** | **0.0028** | 0.0143 | **0.0015** |
| Laterobasal | **0.0002** | **0.0004** | *0.0356* | **0.0008** |
| Centromedial | **0.0028** | *0.0168* | 0.909 | 0.8317 |
| Superficial | *0.0389* | *0.0134* | *0.0155* | 0.0416 |

**HAM-D**

No associations between HAM-D and amygdalar nuclei volumes in MDD survived FDR correction.

**Table S4a.** Correlations for HAM-D and amygdalar volumes in MDD.

| Nuclei/Composite | uncorrected p-value, r-value | |
| --- | --- | --- |
|  | Left | Right |
| Lateral | 0.679, +0.05 | 0.094, +0.20 |
| Basal | 0.914, +0.01 | 0.520, -0.08 |
| Accessory Basal | 0.235, +0.14 | 0.493, +0.08 |
| AAA | 0.260, -0.13 | 0.864, -0.02 |
| Central | 0.58, +0.07 | 0.508, +0.08 |
| Medial | *0.057, +0.22* | *0.005, +0.32* |
| Cortical | 0.266, +0.13 | 0.079, +0.20 |
| CATA | 0.832, +0.03 | *0.033, +0.248* |
| Paralaminar | 0.593, -0.06 | 0.206, -0.15 |
| Whole Amygdala | 0.931, +0.01 | 0.296, +0.12 |
| Laterobasal | 0.403, +0.10 | 0.473, +0.09 |
| Centromedial | 0.118, +0.18 | 0.114, +0.185 |
| Superficial | 0.881, +0.02 | 0.057*, +0.22* |

All results corrected for age, sex and eTIV. Positive or negative r-value denotes positive or negative correlation respectively. FDR was used to correct for multiple comparisons throughout revealing a cutoff for significance of p=0.0019 across the eighteen nuclei and eight composite measures in the MDD group. As such, no value was deemed significant. Italic text denotes p-values <0.05 but not surviving the FDR threshold. Bold text survives FDR correction. AAA, anterior amygdalar area, CATA, cortical amygdalar transition area; eTIV, estimated total intracranial volume; FDR, false discovery rate; HAM-D, Hamilton depression score; MDD, major depressive disorder.

**Table S4b.** HAM-D severity (moderate/severe) and amygdalar volumes in MDD.

| Nuclei/Composite | uncorrected p-value | |
| --- | --- | --- |
|  | Left | Right |
| Lateral | 0.775 | 0.616 |
| Basal | 0.384 | 0.143 |
| Accessory Basal | 0.704 | 0.936 |
| AAA | 0.221 | 0.699 |
| Central | 0.099 | 0.605 |
| Medial | 0.057 | *0.044* |
| Cortical | 0.215 | 0.098 |
| CATA | 0.089 | 0.711 |
| Paralaminar | 0.057 | *0.022* |
| Whole Amygdala | 0.581 | 0.88 |
| Laterobasal | 0.807 | 0.724 |
| Centromedial | *0.023* | 0.227 |
| Superficial | 0.159 | 0.787 |

Univariate analyses of covariance of amygdalar substructures for effects of disease severity. Amygdala volumes from the cohort with moderate illness (HAMD between 17-23, n=52) were compared with severe illness (HAMD≥24, n=28). Age, sex and eTIV were entered as covariates. FDR was used to correct for multiple comparisons revealing a cutoff for significance of p=0.0019 across the eighteen nuclei and eight composite measures in the MDD group. As such, no value survived FDR correction. Italic text denotes p<0.05 but not surviving the FDR threshold. AAA, anterior amygdalar area, CATA, cortical amygdalar transition area; eTIV, estimated total intracranial volume; FDR, false discovery rate; HAM-D, Hamilton depression score; MDD, major depressive disorder.

**Table S5a.** Duration of illness and volumes

Partial correlation showing the relationship between duration and amygdalar subfield volume. Minus sign indicates negative relationship. FDR threshold of p<0.0055. Bold text survives FDR correction. No results survived FDR correction. Italic text denotes p-values <0.05 but not surviving the FDR threshold.

|  | LEFT | RIGHT |
| --- | --- | --- |
| Lateral nucleus |  |  |
| Correlation | 0.102 | 0.148 |
| p-value | 0.378 | 0.204 |
| Basal nucleus |  |  |
| Correlation | -0.001 | -0.012 |
| p-value | 0.994 | 0.916 |
| Accessory basal nucleus |  |  |
| Correlation | 0.077 | 0.085 |
| p-value | 0.507 | 0.47 |
| Anterior amygdaloid area |  |  |
| Correlation | 0.092 | 0.074 |
| p-value | 0.427 | 0.522 |
| Central_nucleus |  |  |
| Correlation | 0.103 | 0.011 |
| p-value | 0.377 | 0.926 |
| Medial_nucleus |  |  |
| Correlation | 0.135 | 0.113 |
| p-value | 0.249 | 0.327 |
| Cortical_nucleus |  |  |
| Correlation | 0.186 | 0.06 |
| p-value | 0.11 | 0.607 |
| Corticoamygdaloid_transitio |  |  |
| Correlation | 0.315 | 0.285 |
| p-value | *0.006* | *0.013* |
| Paralaminar_nucleus |  |  |
| Correlation | -0.145 | -0.164 |
| p-value | 0.213 | 0.157 |
| Whole_amygdala |  |  |
| Correlation | 0.058 | 0.065 |
| p-value | 0.618 | 0.58 |
| Laterobasal |  |  |
| Correlation | 0.125 | 0.034 |
| p-value | 0.278 | 0.774 |
| Centromedial |  |  |
| Correlation | 0.125 | 0.045 |
| p-value | 0.28 | 0.698 |
| Superficial |  |  |
| Correlation | 0.285 | 0.232 |
| p-value | *0.013* | *0.042* |

**Table S5b.** First Presentation Depression (FPD) and Recurrent Depression (RD) groups and volumes.

| Nuclei/Composite | uncorrected p-value | |
| --- | --- | --- |
|  | Left | Right |
| Lateral | 0.863 | 0.323 |
| Basal | 0.622 | 0.613 |
| Accessory Basal | 0.736 | 0.471 |
| AAA | 0.997 | 0.585 |
| Central | 0.807 | 0.216 |
| Medial | 0.162 | 0.584 |
| Cortical | 0.2 | 0.578 |
| CATA | *0.025* | 0.432 |
| Paralaminar | 0.813 | 0.675 |
| Whole Amygdala | 0.469 | 0.399 |
| Laterobasal | 0.633 | 0.472 |
| Centromedial | 0.401 | 0.297 |
| Superficial | *0.050* | 0.216 |

Univariate analyses of covariance of amygdalar substructures for effects of First Presentation Depression (FPD) and Recurrent Depression (RD) groups, based on the number of previous episodes of depression (FPD=0 previous episodes, n=43; RD≥2 previous episodes, n=37). Age, sex and eTIV were entered as covariates. FDR was used to correct for multiple comparisons revealing a cutoff for significance of p=0.0019 across the eighteen nuclei and eight composite measures in the MDD group. As such, no value survived FDR correction. Italic text denotes p<0.05 but not surviving the FDR threshold. AAA, anterior amygdalar area, CATA, cortical amygdalar transition area; eTIV, estimated total intracranial volume; FDR, false discovery rate; HAM-D, Hamilton depression score; MDD, major depressive disorder.

**Table S6.** Medication status and volumes.

Differences between amygdalar volumes between depressed patients on antidepressant medication (87%) and those not on medication at time of scanning. FDR threshold of p<0.0055. Bold text survives FDR correction. Italic text denotes p-values <0.05 but not surviving the FDR threshold. No result survived FDR correction.

|  | LEFT | RIGHT |
| --- | --- | --- |
| Lateral nucleus | 0.184 | 0.064 |
| Basal nucleus | 0.417 | 0.089 |
| Accessory basal nucleus | 0.337 | *0.044* |
| Anterior amygdaloid area | *0.013* | *0.042* |
| Central nucleus | *0.021* | *0.039* |
| Medial nucleus | 0.128 | *0.012* |
| Cortical nucleus | 0.527 | 0.188 |
| Corticoamygdaloid transition area | 0.303 | 0.110 |
| Paralaminar nucleus | 0.396 | 0.937 |
| Whole amygdala | 0.124 | *0.024* |
| Laterobasal | 0.259 | 0.06 |
| Centromedial | *0.029* | *0.011* |
| Superficial | 0.156 | *0.023* |

**Cortisol measures**

Completed cortisol measures across the three timepoints 0, 30 and 60 minutes after waking (T0, T30 and T60) were obtained for 30 MDD patients and 25 HCs. There were no demographic differences between those with a completed cortisol (Table S7).

**Table S7.** Demographic data for cortisol measures.

|  | **Group** |  | **Con v Dep** |
| --- | --- | --- | --- |
|  | **Con N=25** | **MDD N=30** | **p-value** |
| Age: Mean (SEM) | 31.26 (2.1) | 35.12 (2.35) | 0.23 |
| Range (years) | 18-62 | 18-63 |  |
| Male/Female (%M) | 7/18, 37.3% | 10/20, 50% | 0.77 |
| Handedness (R/L) | 23/2 | 29/1 | 1.0 |
| HAMD* (SEM) | 2.3 (0.7) | 23.10 (0.84) | 1.56E-27 |
| MDD months | ---------- | 30 (7) | ---------- |

Con, controls; Dep, depressed; HAM-D, Hamilton Depression scale; M, male; MDD, major depressive disorder; R/L, right or left handed; SEM, standard error of the mean.

There were no significant differences between CAR responses in MDD and HCs (Table S8).

**Table S8.** Group differences for cortisol measures.

| **Cortisol measures**  **(nmol/L)** | **Con (SEM)** | **MDD (SEM)** | **Con vs MDD p-value** |
| --- | --- | --- | --- |
| T0 | 7.57 (0.72) | 11.26 (1.91) | 0.078 |
| T30 | 10.35 (1.09) | 12.77 (1.60) | 0.234 |
| T60 | 7.46 (0.77) | 8.80 (1.41) | 0.433 |
| AUCb | 454.08 (43.03) | 675.69 (114.54) | 0.078 |
| AUCg | 536.02 (46.84) | 685.63 (82.98) | 0.143 |
| AUCi | 81.94 (43.64) | 9.94 (86.22) | 0.486 |

AUCb, area under the curve with respect to baseline; AUCg, area under the curve with respect to ground; AUCi, area under the curve with respect to increase; Con, controls; Dep, depressed; T0, 0 minutes after waking; T30, 30 minutes after waking; T60, 60 minutes after waking. Bold indicates significance at p≤0.05.

No correlations were found for any cortisol timepoint or CAR responses or with any nucleus or composite on any side in the HC group (Table S6). In the MDD group, there was a negative correlation between the left cortical amygdalar transition area at T60 and AUCg (Table 3). There were no correlations for AUCb or AUCi (data not shown).

**Table S9.** Effects of sex on cortisol measures

Differences in cortisol measures between male and female in each group. FDR threshold of p<0.015. Bold text survives FDR correction. Italic text denotes p-values <0.05 but not surviving the FDR threshold. No result survived FDR correction. AUC, area under the curve with respect to ground, AUCi, area under the curve with respect to increase.

|  | CONTROLS | DEPRESSED |
| --- | --- | --- |
| 0 | 0.5453 | *0.043* |
| 30 | 0.736 | 0.7518 |
| 60 | 0.2087 | 0.5348 |
| AUCg | 0.2161 | 0.528 |
| AUCi | 0.6039 | *0.0398* |

**Table S10.** CAR partial correlations in the HC group.

|  | **LEFT** | | | | | |
| --- | --- | --- | --- | --- | --- | --- |
|  | T0 | T30 | T60 | AUCb | AUCg | AUCi |
| Lateral nucleus | 0.044 (-0.443) | *0.038 (-0.455)* | 0.328 (-0.23) | *0.044 (-0.443)* | 0.046 (-0.44) | 0.844 (-0.046) |
| Basanucleus | 0.737 (-0.08) | 0.133 (-0.348) | 0.238 (-0.284) | 0.737 (-0.08) | 0.161 (-0.326) | 0.229 (-0.281) |
| Accessory basal nucleus | 0.545 (0.14) | 0.134 (-0.338) | 0.061 (-0.426) | 0.545 (0.14) | 0.19 (-0.298) | *0.044 (-0.443)* |
| AAA | 0.444 (-0.176) | 0.396 (-0.195) | 0.47 (-0.171) | 0.444 (-0.176) | 0.404 (-0.192) | 0.873 (-0.037) |
| Central nucleus | *0.024* (-0.49) | 0.19 (-0.291) | 0.946 (0.016) | *0.024 (-0.49)* | 0.164 (-0.315) | 0.661 (0.102) |
| Medial nucleus | 0.663 (-0.101) | 0.198 (-0.293) | 0.691 (-0.095) | 0.663 (-0.101) | 0.257 (-0.259) | 0.457 (-0.172) |
| Cortical nucleus | 0.842 (-0.045) | 0.126 (-0.337) | 0.666 (-0.1) | 0.842 (-0.045) | 0.27 (-0.246) | 0.326 (-0.22) |
| CATA | 0.722 (0.082) | 0.168 (-0.312) | 0.886 (-0.034) | 0.722 (0.082) | 0.218 (-0.28) | 0.089 (-0.38) |
| Paralaminar nucleus | 0.812 (-0.055) | *0.033 (-0.466)* | 0.392 (-0.203) | 0.812 (-0.055) | 0.093 (-0.375) | 0.118 (-0.352) |
| Whole amygdala | 0.536 (-0.147) | 0.157 (-0.329) | 0.272 (-0.265) | 0.536 (-0.147) | 0.169 (-0.32) | 0.39 (-0.203) |
| Latero-basal | 0.5 (-0.16) | 0.16 (-0.327) | 0.212 (-0.3) | 0.5 (-0.16) | 0.173 (-0.317) | 0.435 (-0.185) |
| Centromedial | 0.875 (-0.037) | 0.17 (-0.311) | 0.272 (-0.258) | 0.875 (-0.037) | 0.19 (-0.298) | 0.236 (-0.27) |
| Superior | 0.773 (0.067) | 0.197 (-0.293) | 0.386 (-0.205) | 0.773 (0.067) | 0.236 (-0.27) | 0.122 (-0.348) |
|  | **RIGHT** | | | | | |
| Lateral nucleus | 0.277 (-0.255) | 0.653 (0.107) | 0.761 (-0.075) | 0.277 (-0.255) | 0.816 (0.056) | 0.105 (0.373) |
| Basanucleus | 0.424 (0.179) | 0.186 (-0.293) | 0.209 (-0.286) | 0.424 (0.179) | 0.371 (-0.201) | 0.074 (-0.388) |
| Accessory basal nucleus | 0.93 (-0.02) | 0.234 (-0.265) | 0.694 (-0.091) | 0.93 (-0.02) | 0.406 (-0.187) | 0.422 (-0.18) |
| AAA | 0.679 (-0.094) | 0.143 (-0.322) | 0.127 (-0.344) | 0.679 (-0.094) | 0.226 (-0.269) | 0.38 (-0.197) |
| Centranucleus | 0.613 (-0.114) | 0.614 (-0.114) | 0.894 (-0.031) | 0.613 (-0.114) | 0.603 (-0.117) | 0.947 (-0.015) |
| Medianucleus | 0.339 (-0.214) | 0.053 (-0.419) | 0.865 (-0.039) | 0.339 (-0.214) | 0.093 (-0.367) | 0.41 (-0.185) |
| Corticanucleus | *0.03* (-0.486) | 0.086 (-0.394) | 0.283 (-0.26) | *0.03 (-0.486)* | 0.076 (-0.406) | 0.769 (0.07) |
| CATA | 0.68 (-0.093) | 0.174 (-0.301) | 0.836 (-0.048) | 0.68 (-0.093) | 0.232 (-0.266) | 0.387 (-0.194) |
| Paralaminar nucleus | 0.296 (0.233) | 0.314 (-0.225) | 0.15 (-0.326) | 0.296 (0.233) | 0.514 (-0.147) | 0.078 (-0.383) |
| Whole amygdala | 0.306 (0.235) | 0.433 (-0.181) | 0.069 (-0.415) | 0.306 (0.235) | 0.585 (-0.126) | 0.121 (-0.349) |
| Latero-basal | 0.811 (-0.057) | 0.691 (0.095) | 0.816 (-0.057) | 0.811 (-0.057) | 0.714 (0.087) | 0.321 (0.234) |
| Centromedial | 0.417 (-0.182) | 0.213 (-0.277) | 0.861 (-0.041) | 0.417 (-0.182) | 0.251 (-0.255) | 0.669 (-0.097) |
| Superior | 0.497 (-0.153) | 0.101 (-0.358) | 0.6 (-0.121) | 0.497 (-0.153) | 0.157 (-0.313) | 0.406 (-0.186) |

All results reported as ‘p value (r value)’. The FDR threshold was p≤0.000321 for significance. Italic text denotes p-values ≤0.05 but not surviving the FDR threshold. AAA, anterior amygdalar area; AUCg, area under the curve with respect to ground; dof, degrees of freedom; CATA, cortical amygdalar transition area; FDR, false discovery rate; MDD, major depressive disorder. AUCb, area under the curve with respect to base; AUCg , area under the curve with respect to ground; AUCi, area under the curve with respect to increase; dof, degrees of freedom; CATA, cortical amygdalar transition area; FDR, false discovery rate.


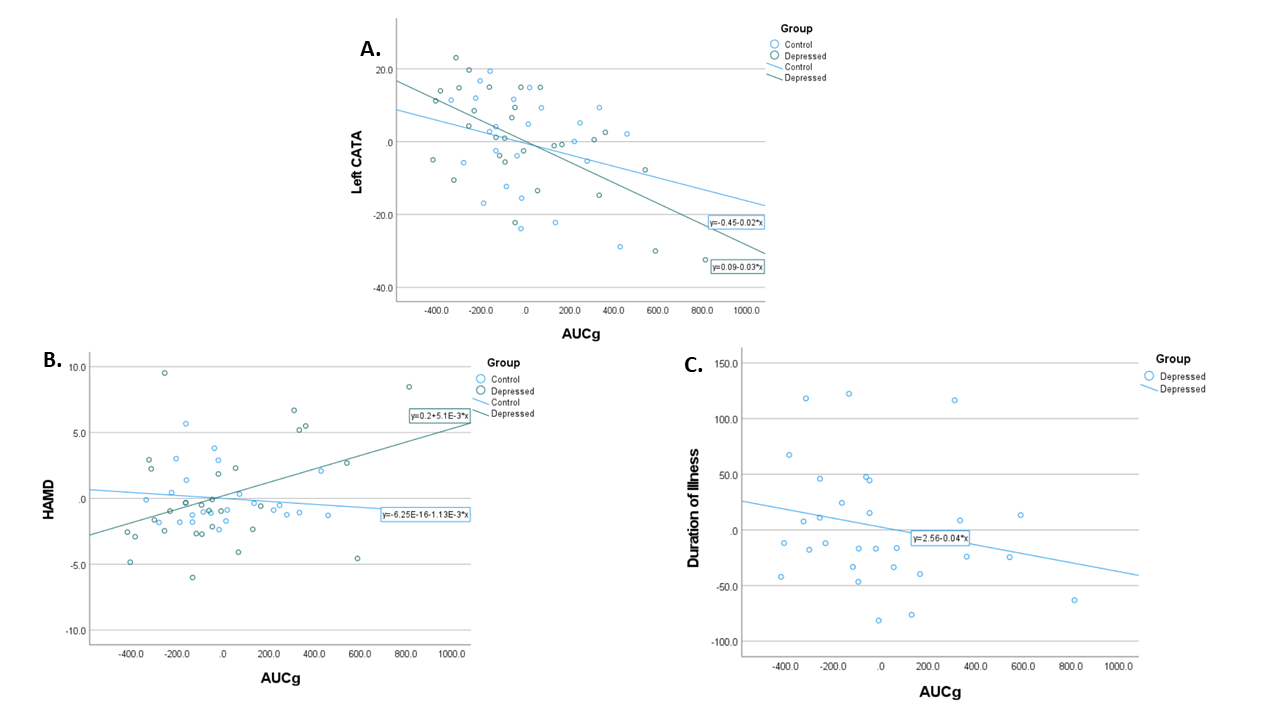


**Figure S1: A.** Scatter plot with residuals from the volume of the left CATA against residuals of AUCg (r = -0.72, p uncorrected 0.00003). **B.** Scatter plot with residuals from the HAMD against residuals of AUCg r = 0.359, p uncorrected = 0.071). **C.** Scatter plot with residuals from the duration of illness against residuals of AUCg in the depressed cohort (r = -0.31, p uncorrected 0.061). All partial correlations were controlled for age, sex and eTIV.

**References**

American Psychiatric Association, 1994. Diagnostic and Statistical Manual of Mental Disorder, 4th ed, Washington, DC.

Benjamini, Y., 2010. Discovering the false discovery rate. Journal of the Royal Statistical Society: series B (statistical methodology) 72, 405-416.

Dale, A.M., Fischl, B., Sereno, M.I., 1999. Cortical surface-based analysis. I. Segmentation and surface reconstruction. Neuroimage 9, 179-194.

Desikan, R.S., Segonne, F., Fischl, B., Quinn, B.T., Dickerson, B.C., Blacker, D., Buckner, R.L., Dale, A.M., Maguire, R.P., Hyman, B.T., Albert, M.S., Killiany, R.J., 2006. An automated labeling system for subdividing the human cerebral cortex on MRI scans into gyral based regions of interest. Neuroimage 31, 968-980.

Doolin, K., Farrell, C., Tozzi, L., Harkin, A., Frodl, T., O’Keane, V., 2017. Diurnal hypothalamic-pituitary-adrenal axis measures and inflammatory marker correlates in major depressive disorder. International journal of molecular sciences 18, 2226.

Fekedulegn, D.B., Andrew, M.E., Burchfiel, C.M., Violanti, J.M., Hartley, T.A., Charles, L.E., Miller, D.B., 2007. Area under the curve and other summary indicators of repeated waking cortisol measurements. Psychosom Med 69, 651-659.

Fischl, B., 2012a. FreeSurfer. Neuroimage 62, 774-781.

Fischl, B., 2012b. FreeSurfer. Neuroimage 62, 774-781.

Fischl, B., Dale, A.M., 2000. Measuring the thickness of the human cerebral cortex from magnetic resonance images. Proc Natl Acad Sci U S A 97, 11050-11055.

Fischl, B., Salat, D.H., Busa, E., Albert, M., Dieterich, M., Haselgrove, C., van der Kouwe, A., Killiany, R., Kennedy, D., Klaveness, S., Montillo, A., Makris, N., Rosen, B., Dale, A.M., 2002. Whole brain segmentation: automated labeling of neuroanatomical structures in the human brain. Neuron 33, 341-355.

Fischl, B., Sereno, M.I., Dale, A.M., 1999. Cortical surface-based analysis. II: Inflation, flattening, and a surface-based coordinate system. Neuroimage 9, 195-207.

Fischl, B., van der Kouwe, A., Destrieux, C., Halgren, E., Segonne, F., Salat, D.H., Busa, E., Seidman, L.J., Goldstein, J., Kennedy, D., Caviness, V., Makris, N., Rosen, B., Dale, A.M., 2004. Automatically parcellating the human cerebral cortex. Cereb Cortex 14, 11-22.

Greenhouse, S.W., Geisser, S., 1959. On methods in the analysis of profile data. Psychometrika 24, 95-112.

Hamilton, M., 1960. A rating scale for depression. J Neurol Neurosurg Psychiatry 23, 56-62.

Heimer, L., De Olmos, J., Alheid, G., Pearson, J., Sakamoto, N., Shinoda, K., Marksteiner, J., Switzer, R., 1999. The human basal forebrain. Part II. The primate nervous system. Part III. Handbook of chemical neuroanatomy 15, 57-226.

Iglesias, J.E., Augustinack, J.C., Nguyen, K., Player, C.M., Player, A., Wright, M., Roy, N., Frosch, M.P., McKee, A.C., Wald, L.L., Fischl, B., Van Leemput, K., 2015. A computational atlas of the hippocampal formation using ex vivo, ultra-high resolution MRI: Application to adaptive segmentation of in vivo MRI. Neuroimage 115, 117-137.

Johnston, J.B., 1923. Further contributions to the study of the evolution of the forebrain. Journal of Comparative Neurology 35, 337-481.

Owen, L.J., Haslam, S., Adaway, J.E., Wood, P., Glenn, C., Keevil, B.G., 2010. A simplified liquid chromatography tandem mass spectrometry assay, using on-line solid-phase extraction, for the quantitation of cortisol in saliva and comparison with a routine DELFIA method. Ann Clin Biochem 47, 131-136.

Reuter, M., Schmansky, N.J., Rosas, H.D., Fischl, B., 2012. Within-subject template estimation for unbiased longitudinal image analysis. Neuroimage 61, 1402-1418.

Saygin, Z., Kliemann, D., Iglesias, J., van der Kouwe, A.J., Boyd, E., Reuter, M., Stevens, A., Van Leemput, K., McKee, A., Frosch, M.P., 2017. High-resolution magnetic resonance imaging reveals nuclei of the human amygdala: manual segmentation to automatic atlas. NeuroImage 155, 370-382.

Sedgwick, P., 2014. Multiple hypothesis testing and Bonferroni’s correction. BMJ : British Medical Journal 349.

Sobie, E.A., 2011. An introduction to MATLAB. Sci. Signal. 4, tr7-tr7.

Van Leemput, K., 2009. Encoding probabilistic brain atlases using Bayesian inference. IEEE Transactions on Medical Imaging 28, 822.

Williams, J.B., 2001. Standardizing the Hamilton Depression Rating Scale: past, present, and future. European Archives of Psychiatry and Clinical Neuroscience 251, 6-12.

Wisse, L.E., Biessels, G.J., Geerlings, M.I., 2014. A critical appraisal of the hippocampal subfield segmentation package in FreeSurfer. Frontiers in aging neuroscience 6, 261.
